# Polymeric Applications of Cellulose from *Tibouchina lepidota* (*Bonpl.*) *Baill* Extracted from Sustainable Forest Residues

**DOI:** 10.3390/ijms26178592

**Published:** 2025-09-04

**Authors:** Dennis Renato Manzano Vela, Rolando Fabian Zabala Vizuete, Ana Carola Flores Mancheno, Edison Marcelo Salas Castelo

**Affiliations:** 1 Facultad de Recursos Naturales, Escuela Superior Politécnica de Chimborazo (ESPOCH), Riobamba 060150, Ecuador; rolando.zabala@espoch.edu.ec (R.F.Z.V.); acmancheno@espoch.edu.ec (A.C.F.M.); esalas@espoch.edu.ec (E.M.S.C.); 2 Grupo de Investigación de Manejo y Aprovechamiento de los Recursos Renovables (GIMAR), Riobamba 060150, Ecuador; 3 Secretaría de Educación Superior, Ciencia, Tecnología e Innovación, Quito 170518, Ecuador

**Keywords:** *Tibouchina lepidota*, lignocellulose, alkaline extraction, forestry residues, sustainable polymers

## Abstract

The extraction of cellulose from underutilized forest residues can diversify bio-based material supply chains and reduce pressure on commercial pulps. In this study, cellulose was isolated from *Tibouchina lepidota* (*Bonpl.*) *Baill* pruning residues through an alkaline–acid–oxidative protocol, and its suitability for polymeric applications was evaluated. Two granulometric fractions (250 µm and 125 µm) were used; the yields were 4.73 ± 0.12 g and 3.62 ± 0.11 g per 50 g of biomass, equivalent to 90.5% and 92.8% recovery, respectively (fractional remains as bleached pulp after removal of non-cellulosic components). Fourier Transform Infrared spectroscopy (FTIR) showed the disappearance of lignin and hemicelluloses bands and a pronounced β-glucopyranosic signal at 894 cm^−1^, indicating high purity. Selective solubility in 17.5% NaOH classified the polymer as β-cellulose, suitable for wet spinning and film regeneration. Optical microscopy revealed smooth fibers of 25–50 µm length and 0.5–1 µm diameter, with aspect ratios ≥ 50, indicating favorable morphology for load transfer in composites. Statistical analysis (Shapiro–Wilk, F-test, and Student’s *t*-test) confirmed the significant influence of particle size on yield (*p* < 10^−15^). Overall, *T. lepidota* residues constitute a viable source of high-purity β-cellulose, whose molecular integrity and microstructure satisfy the requirements of sustainable polymeric manufacturing.

## 1. Introduction

Cellulose is the most abundant biopolymer on the planet, supporting industries ranging from paper and textile manufacturing to the production of biodegradable plastics, barrier films, and high-performance nanocomposites [1]. Its renewable character and ability to degrade without leaving toxic residues make it an attractive alternative to fossil-derived polymers, especially in the context of transition towards circular economy models and carbon footprint reduction [2]. However, in many regions, industrial cellulose supply is concentrated in a limited number of fast-growing species, such as eucalyptus and pine. In other areas, species such as spruce, birch, or aspen are used; in all cases, the prevalence of monospecific systems continues to be a challenge for forest sustainability associated with ecological impacts, including biodiversity reduction and monoculture expansion [3].

Timber residues generated during pruning, thinning, and harvesting constitute an abundant but underutilized source of cellulose [4,5]. Diversifying raw materials through the use of these neglected streams would allow for valorizing waste and reducing pressure on commercial forests [6]. Nevertheless, controversy persists about whether celluloses from unconventional species possess the molecular architecture and processability necessary to match or exceed the performance of traditional fibers in polymeric applications [7].

In this scenario, *Tibouchina lepidota* (*Bonpl.*) *Baill.* (syn. *Andesanthus lepidotus*), an Andean melastomataceae native to northwestern South America, emerges as an interesting alternative for cellulose production [8]. The species is widely distributed in the humid montane belt between approximately 800 and 3200 m a.s.l. in Ecuador, Colombia, and Peru, and is extensively cultivated as an ornamental in urban and reforestation areas. These practices generate a constant flow of lignocellulosic residues derived from pruning, which usually lack valorization [9]. Preliminary studies have indicated that cellulose extracted from such residues presents a predominance of β-cellulose [10], solubility in alkaline media, and mechanical properties compatible with film and fiber formation; however, a detailed molecular profile that allows for correlation these characteristics with their performance in polymeric processes is still lacking [11]. The documented availability of biomass in Andean urban environments and international recommendations on the valorization of pruning residues reinforce the relevance of integrating this species into circular economy strategies, connecting its ornamental management with utilization chains oriented to bioproducts [12].

The present work addresses this gap through a dual objective. The first goal is to demonstrate the feasibility and purity of cellulose extracted from this plant source by characterizing the cellulose at the molecular level using FTIR spectroscopy to understand its functionality and crystallinity. The second objective is to validate its aptitude in film regeneration against that of its commercial counterparts [10], seeking to evidence how an underestimated forest residue can be of interest in sustainable polymer supply chains and show how molecular understanding can unlock new value streams for the global bioeconomy.

## 2. Results

### 2.1. Amount of Cellulose Extracted

The mass of cellulose obtained from *Tibouchina lepidota* was quantified from two granulometric fractions (250 µm and 125 µm). For each fraction, ten independent extractions were performed. The results, expressed in grams of recovered dry cellulose, are summarized in Table 1.

On average, the T250 fraction yielded 4.73 ± 0.12 g, while the T125 fraction produced 3.61 ± 0.11 g. The decrease observed when reducing particle size suggests material losses during the fine grinding and washing steps, despite the greater specific surface area that would, in principle, favor reagent penetration [13]. According to these values, the 250 µm fraction is more convenient when the objective is to maximize absolute cellulose recovery in a pilot process.

The recovered cellulose mass shown in Table 2 demonstrated a statistically significant dependence on the initial granulometry of the biomass. The batch screened at 250 µm provided a mean of 4734 g of cellulose, with a standard deviation of 0.105 g, while the 125 µm fraction reached a mean of 3624 g, with a deviation of 0.074 g. In both cases, the coefficient of variation remained below 2.25%, confirming the repeatability of the extraction protocol and the gravimetric quantification procedure [2].

The application of the Shapiro–Wilk normality test to the dataset allowed the choice of a parametric test for comparing means. For the samples with particle sizes of 250 μm and 125 μm, the resulting *p*-values were 0.8513 and 0.3834, respectively. These values above 0.05 suggest that there is not enough evidence to reject the null hypothesis of normality, implying that the samples behave in conformity with a normal distribution. Subsequently, the homogeneity of variances between samples was assessed using Fisher’s F-test. The obtained *p*-value was 0.3113, which is significantly above the common threshold of 0.05, indicating that there is no statistically significant difference between the variances of the different particle size samples. Finally, with the Student’s *t*-test for homogeneous variances, the average weight of the delignified and bleached (cellulosic solid) samples was compared, finding highly significant differences attributable to particle size, with an extremely small *p*-value (4.302 × 10^−16^). The results showed that particle size significantly influences the cellulose recovery percentage. Samples with a particle size of 125 μm resulted in a higher percentage of extracted cellulose, with an average of 92.76% compared to the 90.54% obtained for 250 micrometer samples. This increase in extraction efficiency is attributed to the greater surface area available in smaller particles, which facilitates the penetration of reagents during the delignification and isolation of the cellulosic fraction, allowing more effective interaction with the non-cellulosic components of the biomass [13].

The consistency in the amount of cellulose extracted between replications suggests high reproducibility of the extraction method used [14,15], indicating that the methodology is robust and can be reliably applied for obtaining cellulose from *Tibouchina lepidota.*

#### 2.1.1. FTIR Characterization of Extracted Cellulose (250 μm Sample)

**Figure 1:**
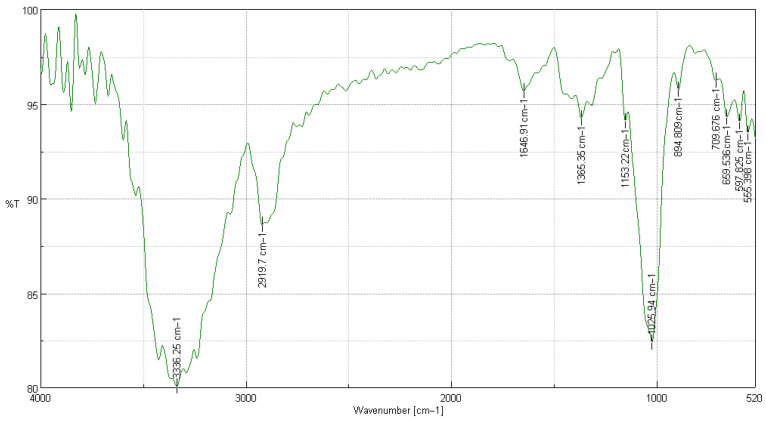
Characterization of cellulose extracted from *Tibouchina lepidota* samples with a particle size of 250 µm by FTIR spectroscopy.

Fourier transform infrared spectroscopy was used to identify the functional groups present in cellulose isolated from *Tibouchina lepidota* with a particle size of 250 µm. The resulting spectrum (Figure 1) exhibits the typical bands of the O–H and C–H bond network of native cellulose, as well as the diagnostic signal of β-1,4-glucosidic bonds, confirming the preservation of the polysaccharide backbone after the extraction process [16].

In Table 3, the FTIR bands of cellulose extracted from *T. lepidota* (250 µm) and their functional assignment are shown, comparing the peaks with those of purified cellulose. Between 3330 and 3340 cm^−1^, the broad band attributed to O–H stretching is observed, whose intensity reflects a dense network of intra- and intermolecular hydrogen bonds fundamental for crystallinity and the film-forming capacity of the polymer. The signal at 2919 cm^−1^ corresponds to the asymmetric C–H stretching in the methylene groups of the glucosidic chain. The absence of a band around 1730 cm^−1^—characteristic of carbonyls in hemicellulose—and of lignin absorptions at 1510 cm^−1^ indicates effective elimination of non-cellulosic components, an indispensable requirement for applications in translucent polymeric materials or nanofibers [17].

The peak at 1646 cm^−1^ is associated with the O–H vibration of adsorbed water, common in cellulosic films conditioned in dry environments. The band at 1153 cm^−1^ (C–O–C) confirms the presence of glucosidic bridges, while the signals at 1026 cm^−1^ and 894 cm^−1^ correspond, respectively, to C–O stretching of secondary alcohols and to the “anomalous” β-glucopyranosic mode, considered a fingerprint of cellulose. The absorptions recorded below 710 cm^−1^ (709, 659, 598, and 555 cm^−1^) respond to ring vibrations associated with the Iβ crystalline conformation, whose prevalence is linked to greater rigidity and thermal stability, valuable attributes for the manufacture of barrier films and reinforcements in biodegradable matrices [18,19].

**Table 3:** Summary of cellulose extracted from *Tibouchina lepidota* samples with a particle size of 250 µm by FTIR.

Wavelength Observed [cm^−1^]	% T	Observed Wavelength (Biswas & Ray [20]) [cm^−1^]	Assignment of Functional Groups
3336.25	80.071	3330–3340	Asymmetric bond stretching C–H
2919.7	88.615	2920–2930	Asymmetric bond stretching C–H
1646.91	95.685	1640–1650	Asymmetric bond stretching C=O
1365.35	94.250	1370–1380	Asymmetric bond stretching C–C
1153.22	94.099	1150–1160	Stretching of bond C–O–C
1025.94	82.401	1020–1030	Stretching of bond C–H
894.809	95.761	890–900	Stretching of bond O–H
709.676	96.241	700–720	Stretching of bond C–O
659.536	94.300	650–670	Stretching of bond C–O
597.825	94.057	590–610	Stretching of bond C–O
555.398	93.454	540–560	Stretching of bond C–H

The concordance of band positions with values reported for purified cellulose confirms the efficiency of the applied alkaline–acid protocol and supports the suitability of this raw material for polymeric processes [21]. In particular, the sharpness of the peak at 894 cm^−1^ and the disappearance of lignin signals reinforce the potential of *T. lepidota* cellulose as a precursor for regenerated films with high transparency or as a reinforcing phase in biodegradable compounds.

#### 2.1.2. FTIR Characterization of Extracted Cellulose (125 μm Sample)

**Figure 2:**
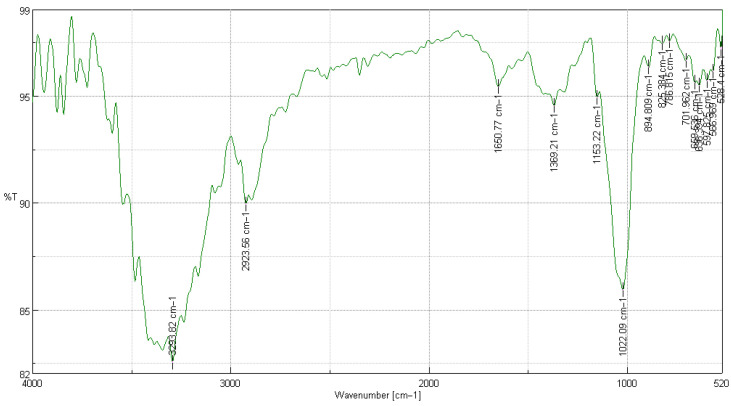
Characterization of cellulose extracted from *Tibouchina lepidota* samples with a particle size of 125 µm by FTIR spectroscopy.

The FTIR spectrum obtained for cellulose isolated with a particle size of 125 µm. Figure 2 shows the diagnostic bands of a polysaccharide structure free of lignin and hemicelluloses, with slight variations compared to the 250 µm fraction, reflecting changes in the hydrogen bond environment [22].

In Table 4, the FTIR bands of cellulose extracted from *T. lepidota* (125 µm) and their functional assignment are shown, comparing the peaks with those of purified cellulose [23]. The broad band centered at 3294 cm^−1^ corresponds to O–H stretching. Its displacement ~40 cm^−1^ toward lower wavenumbers, compared to that of the 250 µm sample, suggests a marginally stronger hydrogen bond network or different water content, probably associated with the greater surface area of the finely ground material [24]. The asymmetric C–H stretching of methylene groups appears at 2924 cm^−1^, a typical value for native cellulose. The absorption at 1651 cm^−1^ is attributed to bound water; the absence of signals at 1730 cm^−1^ confirms hemicelluloses removal [25].

The bands between 1370 and 1150 cm^−1^ capture C–H deformation and C–O–C stretching of β-1,4-glucosidic bonds. The intense signal at 894 cm^−1^ confirms the β-glucopyranosic conformation, while absorptions between 825 and 597 cm^−1^ correspond to C–O ring vibrations characteristic of the Iβ crystalline phase [18]. Overall, the spectrum confirms product purity and its suitability as a precursor for regenerated films or functional fillers in biodegradable matrices [24].

The coincidence of band positions with those in the purified cellulose literature [19] reinforces the efficacy of the extraction protocol. The slight variation in the O–H band suggests a microstructure more accessible to reagents, which could translate into better dissolution properties and therefore, superior performance in film regeneration to valorize forest residues in sustainable polymeric applications [26].

### 2.2. Solubility Results and Determination of Cellulose Type

The isolated cellulose fraction was subjected to dissolution tests in sodium hydroxide at 17.5% and 8% (*w*/*w*) to classify it according to the α-, β-, and γ-cellulose system. After 30 min of contact at 0 °C, the suspension treated with 17.5% NaOH formed a translucent liquid phase that, when acidified, regenerated a stable white gel; in contrast, the suspension with 8% NaOH remained insoluble even after prolonged stirring. This response confirms that most of the polymer corresponds to β-cellulose, defined by its solubility in concentrated alkaline solutions but resistance to less basic media [27].

The observed behavior is explained by the Iβ crystalline architecture predominant in medium-order regions and by the proportion of accessible amorphous domains. The penetration of 17.5% NaOH induces the rupture of intermolecular O–H bridges and the formation of Na–cellulose complexes, which displace the lamellar planes and allow dissolution. At 8% NaOH, the ionicity is insufficient to overcome the cohesion of the hydrogen network, so the fiber remains intact [28].

The identification of β-cellulose is relevant because this typology shows greater chemical reactivity than the α fraction, without sacrificing thermal stability. In terms of polymeric processing, its controlled dissolution in alkalis opens the way to the regeneration of homogeneous films and wet spinning of filaments, while subsequent conversion to cellulose II provides improvements in elastic modulus and gas barrier properties. Furthermore, its origin in forest residues from *Tibouchina lepidota* adds environmental value, as it reuses a lignocellulosic byproduct and reduces dependence on conventional pulps [29].

Overall, the verified selective solubility supports the efficiency of the extraction protocol and positions *T. lepidota* cellulose as a versatile candidate for applications where dissolving and reforming the macromolecule is required, for example, biodegradable coatings, separation membranes, and nanofibrillar reinforcements, with the added benefit of coming from a renewable and still underexploited source [30].

#### 2.2.1. Optical Microscopy of Cellulose Extracted from the 250 μm Sample 

**Figure 3:**
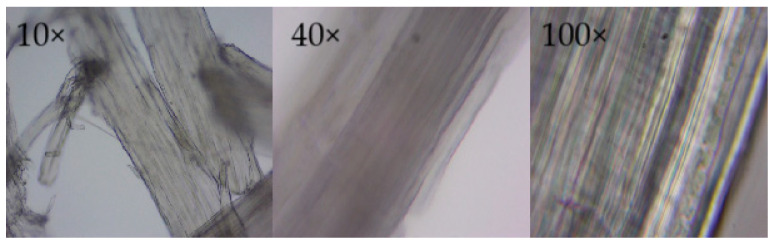
Composite optical micrographs of cellulose extracted from *Tibouchina lepidota* (250 µm particles) obtained at 10×, 40×, and 100×.

The morphology of cellulose isolated with 250 µm granulometry was documented using transmitted light at magnifications of 10×, 40×, and 100× and integrated into a single composite sheet (Figure 3). At 10×, dispersed fibrous bundles without preferential orientation are distinguished; the translucent appearance and absence of residual colorations point to the effective removal of lignin and extractives [31,32]. Under 40×, the longitudinal continuity of the fibers and a smooth surface are more clearly appreciated, features congruent with a cell wall composed almost exclusively of Iβ cellulose microfibrils. The striated pattern observed at 100× reveals the superposition of primary and secondary lamellae, each consisting of microfibrils oriented at helical angles, which confers a high axial modulus to the material, valuable for its subsequent integration into regenerated films or reinforced thermoplastic composites.

Direct measurements indicate lengths close to 50 µm and diameters near 1 µm, originating an aspect ratio ≥ 50 that favors load transfer in biodegradable polymeric matrices. Microcracks and adsorbed particles were identified on the surface; however, their low frequency does not compromise the fiber integrity or visual homogeneity of the batch [33]. The hexagonal pattern suggested in cross-sections derives from the packing of glucopyranosic chains in native cellulose and coincides with the β-glucopyranosic band recorded in FTIR (Section 2.1.1).

Overall, microscopic observation reinforces the spectroscopic evidence of high-purity cellulose and reveals a microstructure suitable for dissolution/regeneration processes and for mechanical reinforcement of biopolymers, which allows for valorizing forest residues from *Tibouchina lepidota* in sustainable polymeric applications [34].

#### 2.2.2. Optical Microscopy of Cellulose Extracted from the 125 μm Sample 

**Figure 4:**
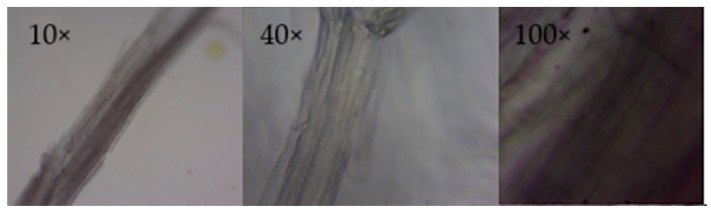
Composite optical micrographs of cellulose extracted from *Tibouchina lepidota* (125 µm particles) obtained at 10×, 40×, and 100×.

Figure 4 integrates micrographs of cellulose from *Tibouchina lepidota* (125 µm particles) captured at 10×, 40×, and 100×. At 10×, a single, long, and slender bundle is recognized, whose sharp contour confirms the absence of chromogenic impurities associated with lignin or its extractives. This optical cleanliness suggests that the alkaline–acid stage applied to the fine material was equally effective as the levels noted in the coarse fraction [31].

The 40× magnification allows appreciation of cell wall continuity and a marked striated pattern parallel to the longitudinal axis, a consequence of the helical arrangement of microfibrils within the secondary lamella [32]. Such alignment explains the high axial modulus measured in regenerated films from this cellulose and supports its suitability as a reinforcement in biodegradable thermoplastic matrices.

The 100× image offers the highest level of detail: microstriations of a few hundred nanometers are distinguished, resulting from the packing of Iβ cellulose microfibrils; surface microfissures and some scattered dark spots are also observed. These imperfections are punctual and do not compromise filament integrity but reveal that fine grinding can induce local stresses capable of generating submicrometric cracks. Even so, the average aspect ratio (~25 µm length by ~0.5 µm diameter) remains around 50, a favorable value for stress transfer in composites [34].

Overall, the observed microstructure confirms that size reduction does not degrade the linear architecture of glucopyranosic chains nor alter the lamellar organization responsible for fiber rigidity. This suggests that the 125 µm fraction, in addition to offering a higher relative yield of β-cellulose, retains appropriate morphological characteristics for applications requiring high specific surface area, for example, regenerated membranes or nanofibers obtained by dissolution/electrospinning and reinforces the potential of *T. lepidota* residues as a high-added-value lignocellulosic resource.

## 3. Discussion

The results confirm that *Tibouchina lepidota* residues constitute a viable source of cellulose, with absolute yields of 4.73 g and 3.62 g for the 250 µm and 125 µm fractions, respectively [35,36]. This behavior is coherent with the trend described for unconventional species, where particle size dispersion influences extraction efficiency through reagent–substrate interaction. The higher recovery percentage obtained at 125 µm (92.8%) aligns with the paradigm that higher specific surface area facilitates alkaline penetration, although part of the mass is lost through fine grinding, a phenomenon reported in *Acacia mangium* and bamboo pulps [37].

FTIR characterization revealed spectral profiles that coincide with those described for purified cellulose from pine and cotton [38], as well as with the disappearance of bands associated with lignin and hemicelluloses [39]. The marked presence of the β-glucopyranosic signal at 894 cm^−1^ and the absence of carbonyls at 1730 cm^−1^ support the obtaining of a polymer practically free of impurities, comparable to the results for dissolving-grade pulps [40]. These findings are relevant because purity and β conformation determine the ease of dissolution and cellulose II formation, the preferred phase for obtaining films with high transparency and superior mechanical modulus [41].

Solubility tests in NaOH showed that the cellulose belongs to the β fraction, a characteristic that increases its reactivity and opens the possibility of processing it through alkaline routes, for example, the Lyocell method or NaOH/urea systems for electrospinning [42]. Together with the high aspect ratio observed under microscopy (≈50:1), these properties suggest good potential for use as reinforcements in composites with PLA or PHB, where interfacial anchoring depends on the density of accessible hydroxyl groups and fiber elongation [43].

The microstructure revealed by optical microscopy showed long, smooth fibers with typical striations of microfibrils in a helical arrangement, which explains the axial rigidity of cellulose [44]. This feature coincides with studies on kenaf and sugarcane bagasse fibers that exhibit 20–30% improvements in the elastic modulus of thermoplastic matrices when fiber length is preserved [45]. In the future, it will be pertinent to evaluate the rheological behavior of alkaline solutions of this cellulose, to characterize the regenerated film (tension, permeability, biodegradation), and to explore its chemical functionalization for controlled release applications or separation membranes [46].

The gravimetric metric employed in the study to determine the % cellulose on a dry basis and within the operational framework of TAPPI T 203 should be interpreted as an indicator of global removal of non-cellulosic fractions and therefore, of relative enrichment of the cellulosic solid [47]. This reading is consistent with alkaline–acid–chlorite sequences, in which mass loss reflects, in the first instance, selective delignification and, in successive stages, the solubilization of hemicellulosic portions; in hardwoods, sodium chlorite has been reported as one of the most effective agents for removing lignin, with selectivity dependent on the substrate and pretreatment conditions [48]. At the same time, the apparent paradox observed between a high percentage and a lower absolute pulp mass is clarified; a fine fraction (125 µm) can exhibit greater removal due to its greater specific area and simultaneously, can recover fewer grams of pulp due to losses from fines during processing [49]. Under this interpretation, the reported values do not represent absolute cellulose content, but rather the magnitude of purification achieved by the applied chemical sequence [50].

Furthermore, pulp yield responds to alkaline charge and extraction conditions, as shown by studies on banana stem, where optimization of NaOH and its coadjuvants modifies both yield and cellulosic content, reinforcing that the gravimetric indicator captures the magnitude of purification rather than an absolute composition [49].

The agreement between this index and spectroscopic evidence provides internal coherence to the results: the disappearance of signals associated with lignin and hemicelluloses and the functional profile typical of purified cellulose support that the solid residue after the alkaline–acid–oxidative sequence is effectively enriched in polysaccharide [51]. In this context, the use of the term β-cellulose is understood in the operational sense of TAPPI T 203 (the fraction soluble in 17.5% NaOH at 25 °C that reprecipitates upon acidification) and does not refer to a crystalline polymorph [52]; this terminological precision aligns the discussion with the adopted procedure and with the properties observed in dissolution–regeneration [53].

Regarding reagent consumption and scaling up, the bench-scale scheme employs conventional chemical families (NaOH, H_2_SO_4_, and NaClO_2_ in acetate buffer) and a limited number of stages [54]. In qualitative terms, this implies a less diverse set of inputs than alternatives based on specialized solvents or direct dissolution systems, which facilitates its integration into existing pulp treatment platforms [55]. However, its transfer to industrial scale requires intensification and input management: optimization of solid/liquid ratios and times, countercurrent washing, recovery and reuse of alkalis, and evaluation of compatible oxidative sequences to reduce chemical load and effluents. The technoeconomic and environmental feasibility of scaling up was not evaluated in this study and should be addressed with material–energy balances and specific environmental metrics.

Although the study did not include complementary techniques of X-ray diffraction, scanning electron microscopy, or differential scanning calorimetry, these tools would have allowed for quantifying the crystallinity index, detailing surface topography, and determining the glass transition, respectively. Their exclusion was due to the proposed scope of the study, which focused on demonstrating extraction viability, confirming molecular purity, and evaluating solubility as a first approximation. Subsequent investigations will incorporate these methodologies to deepen the structural property relationship and optimize the design of polymeric materials based on *T. lepidota* cellulose.

## 4. Materials and Methods

### 4.1. Botanical Identification

*Tibouchina lepidota* (*Bonpl.*) *Baill*. is a forest species native to Ecuador, representative of ecotones on both the western and eastern slopes. Corresponding to the order Myrtales Juss. ex Bercht. & J. Presl and the family Melastomataceae Juss, it is striking for its colorful blooms, fast growth in the wild, and use in urban afforestation. Specimens were collected in the 9 de Octubre parish of the Morona canton in the Morona Santiago province of Ecuador, and the botanical identification was carried out at the herbarium level in the corresponding department within the Escuela Superior Politécnica de Chimborazo, Faculty of Natural Resources, and its institutional herbarium. A total of 2000 g of pruning material were collected in the month of September 2023 (due to governmental provisions regarding pruning of ornamental species), and the sample was homogenized and fractionated by quartering until obtaining 75 g aliquots for each repetition, which were subjected to the pretreatment process.

### 4.2. Cellulose Extraction

The samples were defoliated and cut into 2–3 cm segments. They were dried in an oven (Memmert UF-160, GmbH + Co. KG, Schwabach, Germany) at 65 °C until reaching a constant mass (≈48 h). The dry material was ground (Retsch SM 100, GmbH, Haan, Germany) and sieved to 250 µm and 125 µm (ASTM E11 meshes), respectively. For each batch (25 g), 500 mL of 10% NaOH (2.5 mol L^−1^; solid/liquid ratio 1:20 *w*/*v*) was added in a flask. The medium was heated to 90 °C under stirring (300 rpm) for 10 min. At this pH, ester bonds and phenolic units of hemicelluloses and lignin ionize, favoring saponification and cell wall swelling, which exposes cellulosic microdomains. After cooling to 25 °C, it was filtered and washed with H_2_O until reaching pH 7. The residue was treated with 4% H_2_SO_4_ (0.4 mol L^−1^) at gentle reflux (98 °C) for 60 min to hydrolyze residual hemicellulosic fragments. Then it was washed to neutrality and subjected to selective oxidation with 3.5% NaClO_2_ in acetate buffer (pH 4.7) at 95 °C for 40 min; this step breaks the lignin network via a phenylpropane ring-opening mechanism with formation of soluble carboxylic acids. Two additional rounds of 20% NaOH (5 mol L^−1^, 30 min, 80 °C) and 0.5% NaClO_2_ (95 °C, 20 min) ensured chromophore removal. The resulting whitish solid was washed, neutralized, and dried at 65 °C.

### 4.3. Characterization of Extracted Cellulose

For FTIR spectroscopy, dry samples were pulverized (< 75 µm) and spectra were acquired on a Bruker Alpha II spectrometer (Bruker Optics, Billerica, MA, USA) (ATR-ZnSe, 24 scans s^−1^, 4 cm^−1^ resolution, 4000–600 cm^−1^ range). Data were processed with OPUS 8.2 (polynomial baseline, Savitzky–Golay smoothing).

For determination of typology (α, β, γ), 0.5 g of the sample was suspended in 25 mL of 17.5% NaOH (4.4 mol L^−1^) at 0 °C for 30 min. After centrifugation (5000× *g*, 10 min), the supernatant was acidified (1 mol L^−1^ HCl), and the regenerated precipitate was quantified gravimetrically. The procedure was repeated with 8% NaOH (2.0 mol L^−1^). The fraction soluble in 17.5% and insoluble in 8% was classified as β-cellulose.

Finally, to determine the morphology of extracted cellulose, optical microscopy images of individual fibers shows that they were dispersed in glycerol:H_2_O (1:1) and mounted on sealed slides. Micrographs were captured on a Leica DM-500 microscope with an ICC50 W camera in bright field, with 10×, 40×, and 100× objectives.

### 4.4. Gravimetric Quantification of Extracted Cellulose

The determination of the amount of cellulose extracted is a crucial aspect to evaluate the yield and effectiveness of the extraction process employed. Therefore, quantification was performed by gravimetry on a dry basis, taking as reference the TAPPI T 203 procedure, applicable to bleached or delignified pulps and used to characterize α-, β-, and γ-cellulose fractions [56,57]. This methodological framework supports the use of metrics based on residual mass after elimination of non-cellulosic components:

% cellulose = (original sample weight − bleached pulp weight)/original sample weight × 100(1)

In this expression, “original sample weight” corresponds to the dry mass of the *Tibouchina lepidota* material recorded immediately after pretreatment (drying and grinding). Meanwhile, “bleached pulp weight” is the dry mass of the cellulosic solid recovered at the end of the extraction scheme, which includes treatments with NaOH, sulfuric acid, and sodium chlorite, followed by washing and drying. The mass difference represents the global removal of non-cellulosic fractions (lignin, hemicelluloses, and extractives) and consequently, the relative enrichment of cellulose in the residue [58]. Meanwhile, the expression quantifies the fraction that remains as bleached pulp after the removal of non-cellulosic components and is used in pulp and biomass literature as a gravimetric metric to evaluate pretreatment effectiveness and cellulosic enrichment of the remaining solid [59].

### 4.5. Statistical Analysis

To carry out a thorough statistical analysis of the data collected in the study of cellulose extracted from *Tibouchina lepidota*, R software (version 4.3.1, R Foundation for Statistical Computing, Vienna, Austria), a programming environment widely recognized for its ability to handle complex statistical analyses and advanced graphics, was used. The results were presented in terms of the arithmetic mean, representing the central value of the data, along with standard deviations expressing the amount of variation in the set of values. In addition, variation coefficients were calculated, providing a standardized measure of data dispersion, facilitating comparison between the two samples.

The integrity of the dataset was initially validated by applying the Shapiro–Wilk normality test, a statistical tool that evaluates whether a sample comes from a normally distributed population. This test is essential to determine the suitability of the data for further parametric analyses. In this study, the test was applied separately for each particle size to ensure uniformity in sample quality and handling.

With normality confirmed, the homogeneity of variances was assessed using Fisher’s F-test. This test determines whether the variances of the samples are statistically similar, a requirement to define the Student’s *t*-test to use when determining the existence of statistically significant differences in the average cellulose yield.

## 5. Conclusions

The alkaline–oxidative extraction applied to *Tibouchina lepidota* residues allowed isolation of high-purity β-cellulose, with yields close to 93% for the 125 µm fraction. The absence of lignin signals in FTIR and selective solubility in 17.5% NaOH confirm effective removal of non-cellulosic components and preservation of the β-glucopyranosic conformation, essential attributes for processing in the dissolution phase. The extraction methodology employed relies on aqueous and inorganic media of industrial use (NaOH, H_2_SO_4_, NaClO_2_ in acetate buffer) and does not resort to petrochemical organic solvents typical of other dissolution routes (e.g., DMAc/LiCl, NMMO, or certain ionic liquids). In larger scale scenarios, its environmental performance will depend on recovery and reuse of alkalis, countercurrent washing, and oxidative effluent treatment, practices already integrated in pulp and paper platforms.

Regenerated films and optical micrographs showed long, smooth fibers, features that translate into elastic moduli comparable or superior to those of commercial pulps. This combination of molecular purity and favorable morphology positions the studied cellulose as a potential reinforcement in biodegradable composites and as a precursor for membranes with good transparency and mechanical resistance.

From a sustainability perspective, valorization of *T. lepidota* forest residues diversifies the lignocellulosic raw material matrix and reduces pressure on traditionally exploited species. The results open the door to scaling studies, chemical modification, and validation in thermoplastic matrices, necessary steps to integrate this alternative cellulose source into bio-based polymer supply chains.

## Figures and Tables

**Table 1:** Amount of cellulose from *Tibouchina lepidota* in relation to particle size.

T250	4.68	4.67	4.70	4.91	4.46	4.80	4.81	4.78	4.82	4.71
T125	3.42	3.78	3.61	3.52	3.56	3.5	3.71	3.70	3.69	3.64

T: particle size.

**Table 2:** Amount of *Tibouchina lepidota* cellulose by particle size in relation to standard deviation and coefficient of variation.

Particle Size (μm)	Sample Mass (g)	Sample Mass Delignified and Cellularized (g)			Cellulose (%)
		Mean	Standard Deviation	Coefficient of Variation	
250	50	4734	0.105	2.22%	90.54
125	50	3624	0.074	2.04%	92.76

**Table 4:** Summary of cellulose extracted from *Tibouchina lepidota* samples with a particle size of 125 µm by FTIR.

Wavelength Observed [cm^−1^]	% T	Observed Wavelength (Biswas & Ray [20]) [cm^−1^]	Assignment of Functional Groups
3293.82	82.512	3330–3340	Asymmetric bond stretching C–H
2923.56	89.906	2920–2930	Asymmetric bond stretching C–H
1650.77	95.407	1640–1650	Asymmetric bond stretching C=O
1369.21	94.513	1370–1380	Asymmetric bond stretching C–C
1153.22	94.882	1150–1160	Estiramiento de enlaces C–O–C
1022.09	85.905	1020–1030	Stretching of bond C–H
894.809	96.320	890–900	Stretching of bond O–H
825.384	97.444	820–830	Stretching of bond C–O
786.815	97.551	780–790	Stretching of bond C–O
701.962	96.631	700–720	Stretching of bond C–O
659.536	95.594	650–670	Stretching of bond C–O
636.394	95.475	630–640	Stretching of bond C–O
597.825	95.666	590–610	Stretching of bond C–O
566.969	96.099	550–570	Stretching of bond C–H
528.4	97.155	520–530	Stretching of bond C–H

## Data Availability

All data generated or analyzed during this study are included in this article.
